# Gunshot Wound to the Spine With Delayed Radiculopathy: A Case Report

**DOI:** 10.7759/cureus.32385

**Published:** 2022-12-10

**Authors:** Kunj Patel, Rachita Navara, Faizal Ouedraogo, Ronald Joel, Brock Bowman

**Affiliations:** 1 Physical Medicine and Rehabilitation, Barnes-Jewish West County Hospital, St. Louis, USA; 2 Electrophysiology, University of California San Francisco, San Francisco, USA; 3 Cardiology, Washington University School of Medicine, St. Louis, USA; 4 Medicine, University of Maryland Capital Region Health, Largo, USA; 5 School of Health Professions, University of Missouri, Columbia, USA; 6 Physical Medicine and Rehabilitation, Shepherd Center Hospital, Atlanta, USA

**Keywords:** gunshot wound, case report, spine, decompression, radiculopathy, gunshot

## Abstract

Gunshot wounds (GSWs) to any part of the body can leave a trail of insidious complications. When the spinal cord is the injured organ, these sequelae can be debilitating to the patient and often exhaust all known therapeutic approaches available to the providers. The management of pain associated with GSWs to the spine is often a clinical challenge and there is often a question as to whether or not surgical intervention can help with pain relief in these cases. Here, we present a 45-year-old woman who experienced delayed radicular pain following a GSW to the spine with a retained bullet at the level of the lumbosacral canal. After an unsuccessful comprehensive multimodal analgesia, the patient underwent surgical removal of the bullet, which did not successfully provide a substantial lasting analgesic effect. This case demonstrates the potential for surgical failure and supports the general recommendation of more conservative management in this population.

## Introduction

Gunshot wounds (GSWs) account for 14.3% of spinal cord injuries and can be particularly devastating, leading to a range of neurological deficits [1,2]. The path, size, and type of bullet fired are among the many variables that lead to various levels of patient injury, which range from minimal or no neurologic deficits in about 0.6%, to complete quadriplegia in about 12.4% [1]. Neurological deficits may also progress over time due to either bullet migration in the spinal canal or the formation of a reactive collagenous mass at the site of bullet impact.

Management of GSWs of the spine is, therefore, a clinical challenge, which can lead to devastating complications. Numerous studies represent a consensus in recommending conservative management, as opposed to surgical intervention, as surgical intervention typically does not produce a significant overall benefit, especially when considering complications and recovery issues [3]. Since the majority of these studies are based on GSWs obtained from military wounds, which are often caused by different classes of weapons than GSWs obtained in the civilian population, we need to mitigate some of the findings appropriately [3].

This case describes a clinical case of a civilian GSW to the spine and evaluates the outcome of surgical management.

## Case presentation

**Figure 1:**
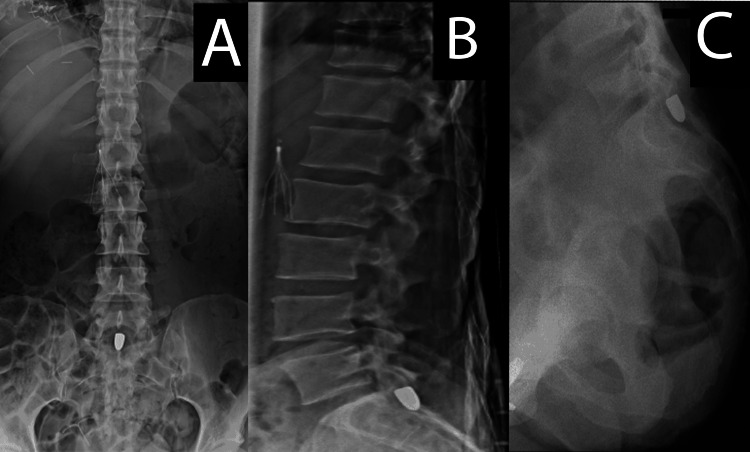
Spinal X-ray (A) Anteroposterior lumbar X-ray, (B) lateral lumbar X-ray, and (C) lateral sacral X-ray. The figure demonstrates the bullet position posterior to the S1 vertebral body.

A 45-year-old woman without personal or familial history sustained multiple GSWs to her torso, bilateral lower extremities, and lower spine. The latter caused an L2 vertebral body fracture before the bullet settled in the center of the spinal canal, posterior to the S1 vertebral body. One month following her injury, the patient developed severe burning left buttock pain, intermittent, worse with movements, which radiated down her left lower extremity to her foot. X-ray imaging showed the bullet had not migrated but appeared to be in a position that could possibly lead to spinal nerve root compression (Figure 1).

Despite escalated doses of gabapentin, tramadol, transdermal buprenorphine, conventional opioid therapy, a left ischial tuberosity injection, and consultation with pain psychology, the patient continued to experience severe uncontrolled pain. Orthopedic and neurological surgery teams were consulted and a laminectomy for bullet removal and nerve root decompression was performed without complications. After surgery, the patient had significant relief from her pain. Two weeks later, the pain returned and once again became unresponsive to medication management.

## Discussion

The outstanding clinical question is whether it is beneficial to perform a surgical intervention to remove bullet fragments and decompress nerve roots after a GSW. The current case demonstrates a bullet removal procedure performed for the intended relief of spinal nerve root compression to the spine that failed to improve the patient’s pain.

There is some support that early decompression improves neurological outcomes after blunt trauma, but there is limited information on decompression after GSW [4]. A study by Bono and Heary in 2004 discussed that undergoing decompression laminectomies for conus or cauda equina level lesions within 72 hours vs. more than 72 hours has some benefits for pain reduction. In particular, the study showed that 47.5% of the patients that underwent decompression surgery within 72 hours had significant improvement and pain reduction [4]. The current patient went more than seven weeks before decompression surgery. The long wait between injury and surgery could be a reason for the refractory pain, despite surgical intervention. Also, given the traumatic nature of the patient’s injury, psychological causes of pain may be highly relevant to her level of pain control, though this was addressed with a pain psychology consultation. Although not relevant to the case at hand, other causes for pain complaints include complications from surgery, migration of bullet fragments, infections, and, rarely, lead toxicity [5].

Often, the decision to manage GSWs conservatively by many physicians stems from concern that surgery has a higher rate of complication [6].

The treatment of pain associated with GSW to the spine is complex and involves many factors including the weapon and type of bullet used, the distance from the firing of the bullet, the trajectory of the bullet, the anatomic location of any bullet fragments, the resultant neurological deficits, the interpretation of imaging, assessment of bullet migration, and the patient’s subjective complaints [3]. Nonetheless, there is very little research into these factors and their relationships to surgical outcomes. Even in cases similar to the case under discussion where the imaging, pathophysiology, and surgical plan all appeared to be consistent with a strong possibility of surgical benefit, the possibility of failure to relieve pain after surgery remains.

In the current case, it is speculated that earlier decompression may have possibly led to a better pain outcome [7]. Additionally, for cases of higher spinal cord injury involvement, a study by Umerani et al. in 2014 demonstrated that 23% of patients who were operated on in less than 24 hours had a > 2-grade improvement on the American Spinal Injury Association (ASIA) impairment scale at a six-month follow-up compared to 8.7% of patients who were operated on after 24 hours [7].

## Conclusions

GSWs to the spine can lead to severe radicular pain. Surgical decompression and bullet removal often fail to provide pain relief despite imaging evidence suggesting nerve root compression is caused by the bullet. Further studies are needed to elucidate the management, particularly the indications and timing of surgery for pain associated with GSW to the spine.
